# Supplementary material for: Are carbon emissions trading and green financial instruments synergistic? -Comprehensive quantitative research based on content analysis
Source: PLoS One. 2024 Mar 7;19(3):e0298601. doi: 10.1371/journal.pone.0298601 (PMC10919601; doi:10.1371/journal.pone.0298601)
Supplement: S1 Data — (ZIP) [file pone.0298601.s001.zip › Data/地级市Z_F工作报告.zip!/║╙▒▒/╟╪╗╩╡║╩╨/└┤╫╘╣1⁄2╓┌║┼í╛┬φ┐╦╩2╛▌═°í┐_2011.docx]

各位代表：

现在，我代表市人民政府向大会作工作报告，请各位代表连同《秦皇岛市国民经济和社会发展第十二个五年规划纲要（草案）》一并审议，并请市政协委员和列席会议的同志提出意见。

“十一五”回顾

“十一五”是秦皇岛发展史上极不平凡的五年。在省委省政府和市委坚强领导下，各级政府同全市人民一道，认真贯彻落实科学发展观，齐心协力、开拓进取，改革开放和现代化建设取得显著成就，市十二届人大三次会议确定的2010年各项任务圆满完成，市十一届人大四次会议通过的“十一五”规划目标全部实现，为“十二五”经济社会发展再上新台阶奠定了良好的基础。

过去五年，我们积极应对国内外环境的复杂变化，克服重重困难，经受住各种严峻考验，办成了一些大事，办好了一些喜事，办妥了一些难事，全市上下发生一系列重要而深刻的变化。我们不断深化对市情的认识，把科学发展、富民强市作为全面建设小康社会的中心任务，全面开启旅游立市战略，统筹生产、生活、生态，为建设“宜居宜业宜游、富庶文明和谐”的新秦皇岛而不懈努力；我们紧紧扭住发展第一要务不放松，做大总量、调优存量，实施一批事关长远的重大项目，有效应对国际金融危机冲击，实现了经济持续平稳较快发展；我们坚定“一中心三基地”产业定位，大力实施节能减排攻坚，积极开展与行业高端、强势企业对标行动，启动创新型城市建设，推动建设以旅游业为中心的现代产业体系，旅游名城建设迈出重要步伐；我们以城镇面貌三年大变样活动为动力，提升城市规划建设管理品质，积极拓展发展空间，塑造滨海城市特色，加快城镇化进程，统筹城乡一体化发展走在了全省前列；我们强化生态优先、环境为要理念，着力加强生态环境保护，努力让秦皇岛的山更绿、水更清、天更蓝、生态更美好；我们出色圆满协办北京奥运足球赛事，充分展示秦皇岛开放、文明、靓丽的崭新形象，留下了宝贵的奥运遗产和永久的奥运印记；我们在保持经济快速发展的同时，更加注重社会建设，千方百计保障和改善民生，建设和谐秦皇岛，人民群众的幸福感不断增强；我们始终把暑期工作作为极其重要的特殊政治任务，高标准完成暑期服务保障任务，做到了环境面貌年年有新改善、服务保障年年有新进步；我们将重大任务、重大活动、重大项目作为干事创业的平台，锻炼干部队伍，检验工作能力，探索形成了一整套行之有效的管理制度和管理模式。秦皇岛五年来的重大变化，既有外在的、有形的，也有内在的、无形的，取得的成绩来之不易，积累的经验弥足珍贵，创造的精神财富影响深远。

五年来，综合经济实力跃上新台阶。2010年，地区生产总值达到930.5亿元，年均增长12％，人均地区生产总值由2005年的16347元提高到31200元；全部财政收入达到140亿元，其中地方一般预算收入达72亿元，分别是2005年的2.5倍和2.8倍。规模以上工业增加值达到283.3亿元，增长1.1倍；全社会固定资产投资完成505.7亿元，增长2.1倍，累计建成千万元以上项目130个，完成投资102亿元。山船重工修造船、中油宝世顺钢管、哈电重装出海口基地、富士康科技园等一批大项目建成投产。社会消费品零售总额达到330.5亿元，增长1.2倍。金融对经济发展的支撑作用不断加强，全市金融机构本外币贷款余额936.5亿元，比2005年增长1.4倍。

五年来，产业结构调整有了新突破。制定并实施重点产业发展规划，产业层次、技术水平和创新能力明显提升。2010年接待国内外游客突破1880万人次，实现旅游总收入147亿元，分别是2005年的1.4倍和2.1倍。服务业增加值437.8亿元，是2005年的1.8倍。规模以上工业企业由488家增加到621家，实现增加值占全部工业的比重达88.6%，提高9.8个百分点。省级以上工程技术研究中心、重点实验室、企业技术中心、博士后工作站达到46家，高新技术企业达到44家。装备制造业、高新技术产业增加值占规模以上工业的比重分别提高1.9个和4.4个百分点。“十一五”节能减排目标如期完成，单位生产总值能耗累计下降20.1%，化学需氧量、二氧化硫排放量累计分别削减16%和12.7%。

五年来，农业农村工作呈现新局面。财政累计投入“三农”资金52.7亿元。各项支农惠农政策全面落实。粮食生产连年获得丰收，2010年总产量达86.8万吨。肉、蛋、奶、蔬菜、果品、水产品产量稳定增长。实施千万元以上农业产业化项目120个，农业产业化经营率达到64.9%。四县地区生产总值年均增长12.6%。完成引青济秦东西线对接工程，戴河、石河等县域“七河”综合整治初见成效。农村生产生活条件不断改善，新增节水灌溉面积37.7万亩，新改建农村公路2850.7公里，行政村全部实现村村通公路、电视、电话。加强以新民居为重点的新农村建设，实施140个省级示范村项目。

五年来，城镇面貌实现新提升。圆满完成三年大变样目标任务，城市现代气息和个性特点愈加鲜明，人居环境更加舒适优美，连续四年在国家城市环境综合整治定量考核中位居河北首位，城镇化率达48.7%。全市森林覆盖率达41.98%，人均公共绿地面积达19.9平方米，城市空气质量二级以上天数保持在351天以上。在省内率先实现污水集中处理县区全覆盖，生活垃圾无害化处理率达100%。城乡规划体系不断完善，城镇空间布局进一步优化，生产力布局更加合理。北戴河新区保护开发纳入省级发展战略。成片改造城中村和老旧小区，累计拆迁700万平方米，建成一批高品质社区。现代立体交通网络进一步健全，群众出行更加便利。城市道路、供气、供热等基础设施日臻完善。山海关古城保护开发等重点项目顺利实施，城市文化内涵进一步凸显。生态景观建设持续加强，城市区“六河”整治一期工程完工，大汤河带状公园、秦皇植物园、北戴河湿地公园等精品工程相继竣工，海岸线景观综合整治、花街花城等绿化美化亮化工程效果明显，北戴河街景整治在全省树立了品牌。四县县城和重点镇面貌实现较大改观，特色更加明显。数字化城管指挥中心建成使用，城市精细化管理水平不断提高。

五年来，改革开放迈出新步伐。国有企业、财税金融、医药卫生、农村、文化等方面的改革深入推进。765户市县区国有中小企业改制任务和耀华集团整体改制工作全面完成。秦港集团实现产权重组和管理体制调整。22家市属事业单位实现转企改制。整合规范投融资平台，成立城市发展投资控股集团。市商业银行增资扩股全面完成。小额贷款公司实现四县全覆盖。民营经济增加值占全市地区生产总值比重达到63%，比2005年提高13.9个百分点。开发区经济总量不断攀升，扩区发展取得重大突破，示范带动作用持续显现。卓有成效地举办了一系列招商活动，一批重大项目签约落地。累计实际利用外资19.6亿美元，到位内资359.9亿元，实现进出口总额183亿美元，年均分别增长16%、21.4%和5.6%。

五年来，民生改善和社会建设取得新进步。财政累计用于民生和社会事业资金205.3亿元，解决了一批群众关心关注的热点、难点问题。城镇居民人均可支配收入由9394元提高到17118元，农民人均纯收入由3376元提高到6214元。城镇累计新增就业22.4万人。城镇养老、医疗等社会保险逐步向灵活就业人员、高校大学生延伸覆盖。四县提前纳入国家新型农村社会养老保险试点，城乡低保标准大幅提高。在全省率先实行廉租住房和经济适用住房保障条件并轨，19427户居民搬入经济适用住房和廉租住房，9692户居民享受了廉租住房补贴。解决了36.32万农村人口饮水或安全问题，6.3万人实现稳定脱贫。城乡免费义务教育全面实现，高中阶段优势教育资源进一步扩大，职业教育毕业生就业率达到96%。医疗卫生体系不断完善，设施技术水平大幅提升，新农合参合率94.3%。乡镇综合文化站、文化信息资源共享、农家书屋等文化惠民工程深入实施，全民健身活动蓬勃开展。“五五”普法工作富有成效。安全生产形势总体平稳，社会和谐稳定，平安秦皇岛建设成效明显，荣获全国综合治理“四连优”称号。国防动员和双拥共建工作扎实开展。审计、统计、人口计生、妇女儿童、老龄、慈善、残疾人、民族宗教、外事侨务、社会公益、气象、人防、地震、地方志、档案等工作取得新成绩。

五年来，政府自身建设得到新加强。以深入学习实践科学发展观、干部作风建设年、创先争优活动为动力，创新政府管理体制机制，全力打造团结务实、廉洁高效政府。新一轮政府机构改革圆满完成。大幅削减市级行政许可和审批项目，行政效能进一步提升。落实重大事项专家咨询、社会公示、听证、质询制度，科学决策、依法行政能力进一步增强。电子政务、政府信息公开和市县乡三级政务服务体系建设走在全省前列。深化行政权力公开透明运行，形成了“机制+科技”防治腐败模式，廉政建设和反腐败工作进一步加强。自觉接受市人大及其常委会的法律监督、工作监督和市政协的民主监督，认真听取民主党派、工商联、无党派人士和人民团体的意见建议，累计办理人大代表建议和政协委员提案2396件，办复率100%，满意率达98%以上。

2010年是“十一五”的收官年，也是承上启下的关键年，经济社会发展呈现许多新的亮点：一是实现经济发展速度、质量、效益同步提升。地区生产总值比上年增长12.3%，全部财政收入、地方一般预算收入分别增长22.4%和27.9%。城镇居民可支配收入、农民人均纯收入分别增长10.7%和12.7%。二是凝聚形成旅游立市的高度共识。成立旅游发展委员会，健全政府主导、部门联动、市场运作、社会参与的大旅游发展机制；实施旅游立市百项工程，全方位发展旅游、全产业融合旅游的发展格局初见成效。三是赢得系列难得的重大战略机遇。我市被确定为全国首批服务业综合改革试点、首批旅游综合改革试点城市。四是在服务暑期、推动发展上实现新突破。暑期服务保障的思路更加清晰、措施更加务实，上级关注更高、支持更大。暑期工作成为引领各项事业提质量、上水平的重要平台。五是实施一批破解瓶颈制约的重大交通基础设施项目。津秦客专、承秦高速、城市西部快速路等重点项目加速推进，北戴河机场、秦皇岛和北戴河火车站改造开工建设。六是办了一批直接关系人民群众切身利益的实事。

各位代表！过去五年成就的取得，得益于改革开放多年来奠定的良好基础，是省委省政府和市委正确领导的结果，是市人大、市政协及社会各界大力支持的结果，是五年来市政府历任组成人员团结奋斗，各县区、各部门和全市人民共同努力的结果。在战胜困难与挑战，完成各项重大任务中，充分体现了全市人民和衷共济、一往无前的决心、智慧和勇气。在此，我代表市人民政府向全市人民、人大代表、政协委员，向各民主党派、工商联、无党派人士和人民团体，向驻秦人民解放军、武警官兵和公安政法干警，向中省直机关驻秦各单位，向关心支持秦皇岛发展的海内外朋友们，致以崇高的敬意和衷心的感谢！

“十二五”展望

各位代表，“十二五”是我市加快转变经济发展方式、建设沿海强市的重要时期。

综观国内外发展大势，后危机时代全球经济结构调整步伐加快，国内南资北移趋势明显，经济结构转型加速，环渤海经济圈和京津冀地区快速崛起，河北沿海地区开发建设即将纳入国家发展战略，我市被列为全国首批服务业和旅游综合改革双重试点，各级各方面对秦皇岛发展的期待更多、支持更大、要求更高，我们迎来了多载难逢、大有可为的重要战略机遇期。与此同时，我们也面临诸多困难和严峻挑战。全球经济环境更趋复杂，国家宏观调控不确定性因素增多，区域和城市间竞争日趋激烈，制约我市加快发展的体制机制障碍依然较多，经济总量不够大、主导产业不够突出、城乡发展不够均衡、发展空间不够足等深层次矛盾和问题日益凸显，我们面临的压力更大、任务更加艰巨，要求我们思想解放力度、工作力度、创新力度必须有大的突破和跃升。

面对省委省政府将我市列入沿海地区跨越发展第一梯队的殷切厚望，反思秦皇岛与先进地区的差距，感受全市人民追求美好生活的强烈愿望，我们必须不断强化机遇意识、使命意识、责任意识，唱响“科学发展、富民强市”主旋律，努力在新一轮更好更快发展中抢占先机、赢得主动，争当河北科学发展排头兵和展示形象重要窗口。

按照中共秦皇岛市委十届七次全会精神，我市“十二五”经济社会发展的总体要求是：认真贯彻党的十七届五中全会精神，全面落实科学发展观，突出科学发展主题和加快转变经济发展方式主线，围绕加快发展、加速转型双重任务，统筹生产生活生态，大力实施旅游立市战略，着力调整优化产业结构，着力加快城乡统筹发展进程，着力推进改革开放创新，着力加强人文生态建设，着力保障和改善民生，扎实推进经济社会更好更快发展，全力打造全国现代服务业先行区、全国生态文明先行区，建设“宜居宜业宜游、富庶文明和谐”的滨海名城。

综合考虑各方面因素，并与全面建设小康社会目标相衔接，“十二五”时期经济社会发展的主要目标是：“一个率先、三个翻番、五个突破”。即2014年在河北率先达到全面小康社会水平，人均地区生产总值比2000年翻两番；力争“十二五”末，地区生产总值比2010年翻一番，地方一般预算收入翻一番，固定资产投资翻一番半；城镇居民人均可支配收入突破3万元，农民人均纯收入突破1万元，科技进步对经济发展的贡献率突破60%，城镇化率突破58%，森林覆盖率突破44.5%。

实现上述目标要求，关键是立足当前，着眼长远，统筹兼顾，突出重点，以追求更好的转型发展、更快的提速发展、统筹城乡的协调发展、开拓进取的创新发展、“人文+生态”的绿色发展、人民幸福的和谐发展，带动秦皇岛在未来五年实现跨越崛起。

（一）以旅游名城建设为总揽，实现转型发展。强化大旅游和综合性产业观念，推进各产业与旅游元素的有机融合，以旅游的大发展带动各产业向更高层次迈进，向具有秦皇岛特色的现代产业体系转型，打造“一中心三基地”，同时加快用先进适用技术改造传统优势产业，以“六化”建设为统领大力发展现代农业，积极发展海洋经济和新兴服务业态。一是推进旅游业转型升级，优化全市域“两带六区”空间布局，抓好休闲城市服务标准化建设，以旅游立市百项工程为支撑，北戴河新区为突破口，开发高端旅游产品，缓解淡旺季矛盾，促进旅游业从观光游向观光与休闲度假游并重转变，实现旅游总收入翻一番以上。二是做大做强以装备制造业为主的先进制造业，集群化打造400万吨造船基地、重型装备出海口基地、大型工程装备制造基地、高速铁路设备生产基地、世界级轮毂生产基地。三是发展壮大以高新技术产业为主体的战略新兴产业，以电子信息、节能环保、生物医药等为重点，积极引进一批高技术项目，培育产业集群，推动高新技术产业加快成长为我市先导性、支柱性产业，战略性新兴产业增加值占地区生产总值比重达到15%以上。四是加快实施西港东迁，完善港口功能，优化岸线布局，拓展腹地空间，建设国际型、现代化、综合性大港，打造服务“三北”的重要物流节点城市，集装箱吞吐量达100万标箱。

（二）以项目园区建设为突破口，实现提速发展。把项目园区建设摆在经济发展的重中之重位置，着力引进大项目、建设大平台、培育大企业，千方百计扩充总量，提升实力，让转型发展建立在快速发展的基础之上。以北戴河新区、开发区扩区、西部工业区、大北部工业区、山海关临港产业聚集区等为重点，强化政策资金支持，创新体制机制，完善基础设施和公共服务配套设施，促进投资向园区集聚、企业向园区集中，实现一年一大步、五年大跨越，打造新的战略增长极。力争到2015年，开工建设总投资50亿元、百亿元重大项目各6个以上，培育销售收入超50亿元的企业集团10家、超100亿元的企业集团5家，培育主营业务收入超500亿元的产业聚集区2个以上，超千亿元的产业聚集区1个以上。

（三）以城乡一体化为路径，实现协调发展。坚持把城镇化作为统筹城乡发展的重要抓手，以城带乡、以工促农，打造城乡建设布局科学、经济要素高效流通、生活空间相互融合、项目设施共建共享、公共服务均衡同质的城乡一体化新格局。一是着眼推动城市环境质量、聚集能力、承载功能、居住条件、风貌特色、管理服务再上新台阶，实施城镇建设三年上水平十大工程，打造精品、塑造特色、彰显魅力，在更高起点上提高城市规划建设管理水平。二是推进交通基础设施、公共服务和城市文明向农村延伸，打造市域半小时交通圈、20分钟城乡互动生活圈。三是推进县城、重点镇扩容升级，依托园区（产业聚集区），加快农村二、三产业发展，促进人口向城镇集中，打造优美县城和特色中心镇。四是稳步推进农村新民居建设，着力改善农村居民住房条件，推动新农村建设取得重大进展。

（四）以改革开放创新为动力，实现创新发展。把改革开放创新作为加快转变经济发展方式的重要支撑，激发潜在活力、增强内生动力、提高核心竞争力，争创四方面发展新优势。一是扎实推进乡镇机构改革和行政审批、医药卫生、农村综合配套等重点领域和关键环节的改革，突出抓好全国服务业、旅游两项综合改革试点，完善有利于科学发展的体制机制，发挥大胆探索、敢于突破的先行先试优势。二是消除制约民间投资的制度性障碍，放手、放胆发展民营经济，推进全民创业，壮大市场主体，力争民营经济增加值翻一番以上，形成民间投资充分涌流的后发优势。三是统筹对内对外开放，外资、外经、外贸并举，资金、项目、人才、技术并重，创新招商方式，增强招商实效，着力引进重大战略投资者，全面提升开放型经济层次和水平，实际利用外资、引进内资年均增长10%和15%，进一步放大首批沿海开放城市的基础优势。四是积极开展以科技创新为重点的创新型城市建设，打造以优质或高附加值产品为主导的产业体系，以企业为主体、市场为导向、产学研紧密结合的技术创新体系，以高精尖人才为引领、各级各类人才均衡发展的人才体系，新建企业技术研发机构30家以上，培育拥有自主创新能力和品牌的企业50家以上，万人专利申请量达8件以上，全面提升既有产业和科研方面的比较优势。

（五）以人文生态建设为抓手，实现绿色发展。把人文作为城市底蕴来重点培育，生态作为城市魅力来重点打造，全面提升城市内在功能品质和外在容貌形象。一方面，围绕提升发展软实力，深入挖掘和传承传统特色文化，创作文化精品，培育具有开放性、包容性的地域特色文化品牌和城市人文精神；推进文化体制机制改革，培育一批具有较强影响力的文化旅游企业集团；加快实施文化惠民工程，建设覆盖城乡的文化服务体系；制定并落实文化产业发展纲要，发展以文化创意为核心的文化产业，促进文化产业与各产业的深度融合，力争文化产业增加值占地区生产总值比重达8%以上。另一方面，围绕打造全国生态文明先行区，持续不断地开展大规模、高层次的城乡造林绿化和生态景观建设，深入实施“蓝天碧海金沙行动”，建设北方花城和生态水城，打造国家森林城市；加强环境友好型、资源节约型社会建设，推行绿色清洁生产模式和消费方式，推动节能减排和绿色低碳发展，确保单位生产总值能耗降低15%、二氧化碳排放量降低15%，主要污染物排放总量减少10%。

（六）以保障和改善民生为目的，实现和谐发展。坚持以人为本、民生为重，将更多的新增财力用于社会建设和民生改善。围绕就业再就业、城乡社会保障、社会建设管理和平安秦皇岛建设4项重点，以及住房、就医、教育等难题，切实办好直接关系群众生活的实事，不断提高社会管理和公共服务水平。力争到2015年，新增城镇就业23万人，实现农村劳动力稳定充分转移就业，居民养老保险全覆盖，城镇低收入困难家庭住房实现应保尽保，城市社区卫生服务街道覆盖率达100%，新农合参合率达95%以上；教育发展主要指标高于全省平均水平；安全生产、食品安全、社会安全持续稳定，暑期服务保障水平逐年提高，人民幸福感不断增强。

各位代表！把秦皇岛的明天建设得更加美好是全市人民的共同期盼。我们已经打下了一个良好的发展基础，也一定能够谱写更加辉煌的新篇章！我们相信，经过全市上下五年的努力奋斗，一个经济更加繁荣、社会更加和谐、城市更加靓丽、环境更加优美、百姓更加富足的秦皇岛必将展现在世人面前！

开局之年主要任务

今年是中国共产党成立90周年，也是“十二五”开局之年。我们将紧紧围绕科学发展主题和加快转变经济发展方式主线，把主要精力集中到“稳增长、调结构、控物价、惠民生”上来，确保“十二五”开好局、起好步。

经济社会发展主要预期目标是：地区生产总值增长12%左右；全部财政收入增长12%以上；规模以上工业增加值增长15%；全社会固定资产投资增长20%以上；社会消费品零售总额增长18%；外贸出口总额增长8%；城镇居民人均可支配收入和农民人均纯收入增长12%左右；单位生产总值能耗和二氧化碳排放量均下降3%，化学需氧量、二氧化硫和氨氮、氮氧化物排放量均削减1.5%；登记失业率控制在4%以内；居民消费价格总水平上涨4%左右；人口自然增长率控制在6.3‰以内。上述目标，是基于当前形势和发展走势，对全市经济社会发展提出的指导性、约束性指标。沿海各县区、开发区及各园区（产业聚集区），要全力打好沿海地区开发建设“九大攻坚战”，在实际发展中务求更好、更快。

（一）积极扩大内需，保持经济平稳较快发展。一是大力加强项目建设。实施千万元以上在建项目360个以上，年计划投资464亿元以上，增长20%。其中，亿元以上新开工60个以上、投产20个以上。全市谋划储备亿元以上重大项目1000个以上，各县区、开发区、北戴河新区储备重点项目各500个以上，争取更多列入省、国家“十二五”规划和年度计划。优化投资结构，工业投资、服务业投资分别完成180亿元和397亿元以上，占全社会固定资产投资比重分别达29.6%和65.4%。二是着力抓好经济运行调节。突出主导产业和新兴产业的运行监控，建立生产要素保障联动机制，确保重点企业生产资料供应。加强县区工业经济综合评价，实施市、县、乡“一对一、点对点”对标帮扶，力争主营业务收入超10亿元、超50亿元企业分别达30家、5家。三是努力扩大消费需求。全面落实事业单位绩效工资制度，推进非公有制企业职工工资集体协商机制，落实低收入群体发放临时价格补贴机制，提高居民消费能力。加强社区服务中心及配套公共设施建设，打造便民利民的“十分钟生活圈”。大力发展假日经济和夜经济，培育新的消费热点。落实房地产市场调控各项政策，促进房地产市场平稳健康发展。全面落实“家电下乡”、“家电以旧换新”工程各类补贴政策，扩大农村消费需求。加强市场价格预警监管，确保市场价格基本稳定。

（二）抓好重点区域开发建设，着力培育新的战略增长极。一是全力推进园区（产业聚集区）发展。开展园区（产业聚集区）建设攻坚年活动。6月底前全部完成产业聚集区发展规划审批工作。加大基础设施、研发中心等公共服务设施建设力度，年底前所有产业聚集区达到“九通一平”。全面落实省支持产业聚集区发展的优惠政策，细化具体措施，增强对大项目、大企业大集团的吸引力。积极探索园区（产业聚集区）市场化开发的新路子。力争全市园区（产业聚集区）固定资产投资增长30%以上，主营业务收入突破1100亿元。二是加快北戴河新区开发建设。科学规划利用岸线资源，优化开发时序，坚决杜绝引进建设与定位相悖的项目。加快路网、智能电网、公用配套设施建设，实施沿海公路拓宽改造一期、新区主干路、综合旅游码头等工程，6月底前完成抚南连接线、昌黄连接线道路及景观建设。加快重点产业支撑项目建设，暑期前生态休闲浴场等项目投入使用，争取城市综合体、恒大金碧天下等7个项目年内开工建设。积极引进战略投资者，争取更多高端项目落地，切实把新区建设成全市产业结构调整优化的新引擎。三是高标准推进开发区扩区发展。科学制定扩区开发总体规划。适度超前开展基础设施建设。围绕电子信息、生物医药、现代服务业、装备制造业等主导产业，加快中信戴卡基地、诺贝尔生物医药基地等项目建设，争取引进2－3家超亿美元的重大项目，培育特色鲜明的新兴产业聚集区和统筹城乡发展示范区。

（三）加快发展服务业，倾力打造全国现代服务业发展先行区。一是全面开展国家综合改革双重试点工作。统筹制定实施方案，创新管理体制机制以及相关政策措施，全力探索以旅游业为龙头的现代服务业发展路径，努力为全国同类地区发展提供示范。二是推动旅游业加速转型。按照5A级景区标准，完成全域发展战略规划。加快北戴河五星级酒店等项目建设进度，暑期前争取形成一批新看点、新亮点。大力改造提升既有景区，暑期前全市A级景区达到30家以上。抓紧谋划实施休闲度假、休疗养生、大型演出等系列项目，力促北戴河国际旅游度假中心、抚宁葡萄岛等项目尽快落地开工。开展旅游环境提升年活动，实施旅游景区基础设施建设项目30个以上；充分运用国家城市休闲服务标准化试点验证成果，完善涉旅行业服务规范及质量评价体系。积极拓展国内外旅游客源市场，打响“长城滨海画廊、四季休闲天堂”品牌。三是着力提升现代物流业。协调加快西港区煤炭退出，支持秦港集团按照“物流港”理念规划建设各港区，发展集装箱业务。推进秦皇岛港临港物流、山海关临港物流、出口加工区保税物流、空港物流等物流园区建设，抓好兴龙仓储物流中心、福凯物流服务园等项目建设，培育发展更多物流主体。谋划建设内陆无水港，拓展腹地空间。四是积极发展文化产业。完善北戴河创意产业园区等文化产业片区，加快动漫产业园等文化产业项目建设，支持建设酒吧、艺术品等特色街区，促进文化创意、动漫游戏等产业尽快形成规模，培育文化品牌。五是培育发展新兴服务业。优化金融生态环境，支持各类金融机构发展，积极引进金融机构，鼓励发展新型农村金融组织，力争全年新增贷款140亿元。加快发展社会化养老服务业。积极培育总部经济、商务会展等新兴业态，推进万科会展中心、北戴河国家科技展览中心建设，全力协助科技部办好第一届北戴河全国科技成果展。争取我市成为国家服务外包试点城市。

（四）大力调整优化产业结构，提高经济发展质量和水平。一是壮大装备制造业。重点抓好山船重工造船设施及修船扩建、首秦公司钢材深加工二期工程等续建项目，开工建设天启数控加工中心、启新水泥生产线除尘装置等项目，争取光宇集团电动汽车、金轮发电机组等项目尽快落地，推动装备制造业向规模化、集群化、高端化发展。二是改造提升传统优势产业。加快信息化与工业化深度融合，大力推进技术改造，实施重大技改项目50项。抓好首钢板材公司退城进郊、耀华高新技术产业园区、金海粮油物流中心等项目建设，加快金属压延业、玻璃工业、粮油食品业的产业链拓展和价值链提升。实施华夏长城葡萄酒扩建、柳河酒庄群等项目，推进产业结构优化和企业整合，力促干红葡萄酒产业规范健康发展。三是积极培育以高新技术产业为主体的战略性新兴产业。设立专项资金，培育发展电子信息、节能环保、生物医药等战略性新兴产业。抓好燕大捷康特电子射频识别系统、康泰医学医疗器械技改等高新技术产业项目，加快数据产业基地和北戴河信息技术研发中心建设。争取高新技术产业增加值增长18%。四是抓好节能减排。落实省“双三十”示范工程。以年综合耗能3000吨标煤以上企业为重点，开展能效水平对标活动。对重点排污企业实施全程监控。新建北戴河新区、北部工业区污水处理厂，升级改造市第三污水处理厂，启动省级重点镇污水处理厂建设，新增污水日处理能力14万吨。强制推行玻璃等工业企业煤改气，有序淘汰“三高一低”企业。争创国家级环保模范城市。五是加快科技创新步伐。争列国家创新型城市试点。新建工程技术研究中心、企业技术中心5家，建设国家级科技企业孵化器1家，研发、引进优质新产品100个。推进数据产业研究院和燕山大学国际科技合作基地建设，抓好天业通联国家创新方法试点创建工作。落实人才发展规划纲要，统筹抓好城乡人才开发，启动建设以公共租赁房为主体的“人才家园”，着力培养、引进高精尖人才。

（五）加速城镇化进程，推动城镇建设上水平、出品位。一是加强基础设施建设。暑期前完成北戴河火车站改造、西部快速路工程。年底前完成秦皇岛火车站站前广场改造和秦抚快速路主体工程，津秦客专建设完成，北戴河机场完成工程总量的60%，加快承秦高速、滨海公路建设进程，启动建设卢昌快速路；完成北戴河西部水厂及配套管网建设；实施北戴河新区天然气管道、秦沈长输管道天然气等工程；加快热电联网二期集中供热工程，新增集中供热面积300万平方米。二是塑造独特形象魅力。加快金梦海湾、火车站周边、归提寨以及北戴河杨各庄等片区改造建设，推动海港区北部新城区、北戴河草厂片区开发建设，打造高品位城市功能区。暑期前建成北戴河海上音乐厅、迎宾馆，完成北戴河亮化以及滨海大道、龙源大道、西环路等景观提升工程，打造北戴河联峰路、山海关一关路等一批新亮点。年底前完成海港区开滦路历史文化街区改造一期主体工程、青龙满族风情街建设，启动卢龙古城保护开发部分工程，丰富城市文化内涵。抓好县城和重点镇建设，打造抚宁县城文体中心、昌黎县城东山新天地、卢龙县城阳光水岸小区、青龙满族自治县城迎宾路十里景观带等一批形象提升工程。三是巩固生态环境优势。暑期前完成北戴河森林湿地公园二期等工程建设，人工造林12.48万亩，新增城市园林绿地410万平方米，人均公共绿地面积达到20.3平方米。实施花海建设工程，塑造更高品位的“花街花城”。加快“生态水城”建设，实施城区“六河”水系治理二期工程，持续加强县域“七河”水系治理，实现戴河与新河水系沟通连接。抓好海水污染防治和海滩恢复治理。四是提升城镇管理水平。高标准抓好城乡规划体系建设，确保经济社会发展站在高品质的规划基础之上。实质性推进城市区与抚宁、昌黎县城区域一体化管理。完善城市管理数字化平台，提升城市精细化、标准化、信息化管理水平。加大以交通秩序、景区与车站周边环境等为重点的综合整治力度，改善城市运行秩序。深入推进全国文明城市和文明景区创建工作。

（六）统筹城乡发展，加快建设社会主义新农村。一是提高农业综合生产能力。稳定粮食播种面积，提高单产水平。抓好“菜篮子”建设，全年蔬菜、肉、蛋、奶、果品、水产品产量分别达到350万吨、32万吨、12.5万吨、11.5万吨、77万吨和40万吨左右。实施优种工程和科技进村入户工程，主要农产品生产基地标准化生产普及率达到90%以上。加快水利改革发展，大力加强水利基础设施建设，改建加固堤防30公里，除险加固病险水库17座，新建水池5000个，新增节水灌溉面积3万亩，切实增强水利支撑保障能力。二是推进农业产业化经营。实施千万元以上农产品加工项目50个以上，完成投资15亿元，农业产业化经营率达到65.4%。加快正大生猪产业化、青龙中红三融肉鸡屠宰深加工等重点项目建设，壮大粮油加工、生猪、肉鸡、海洋水产等主导产业规模。健全农产品市场体系和社会化服务体系，新增农民专业合作社100家。三是加快壮大县域经济。抓好抚宁杜庄工业聚集区、昌黎工业聚集区、卢龙龙城工业区、青龙工业聚集区以及卢昌工业园建设，确保年营业收入增长20%以上。加快发展品牌农业、外向型农业、生态观光农业、庄园经济及沟域经济，每个县区实施1－2个大型农业综合开发示范项目。大力发展劳务经济，力争全年劳务经济总量达到43亿元以上。四是稳步抓好农村新民居建设。坚持因地制宜、分类指导、尊重民意，启动100个村以上的建设工作。统筹新民居与创建文明生态村为主要内容的农村基础设施建设，新建农村公路94.5公里、户用沼气池4000个，解决7万人的饮水安全问题。积极引导农村土地承包经营权流转，发展适度规模经营，力争流转面积占承包土地总面积的比重提高1.5个百分点以上。全面建立村级集体财富积累机制，增强村级组织运转保障能力。

（七）深化改革、扩大开放，增强经济社会发展的动力和活力。一是推进行政审批制度改革。最大限度地把相关审批权下放到各县区。对符合产业定位的项目，实行地价减让、规费减免、财政扶持等优惠政策，对重大项目实行全市统筹、一事一议。理顺园区（产业聚集区）管理体制，推行扁平化管理。二是强化国有资产运营管理。进一步完善国有资产监管体制。支持帮助耀华集团完成内部资源整合，建立现代企业制度。加快奥莱特腈纶公司破产重整、新港港务公司资产整合和中阿化肥公司一、二期资产及复合肥产业链整合。提高国有资本运营质量，促进国有资产保值增值。三是发展壮大民营经济。全面落实民营经济发展各项优惠政策，拓展民营资本发展空间，促进民间投资快速增长。市、县区政府安排专项资金，加大对中小企业发展的支持力度，加快融资性担保体系建设，支持民间资本参与发起设立村镇银行、小额贷款公司等融资服务机构。鼓励符合条件的企业上市融资。支持秦皇岛丰收企业发展促进中心扩大服务范围。鼓励全民创业，力争民营经济增加值增长15%，主营业务超亿元企业达到120家。四是增强招商引资实效。实施更有效的招商引资和对外开放激励政策，加强招商队伍建设，推行招商工作人员聘任制、绩效工资制，推行驻地、委托等有效招商方式。充分发挥各级领导特别是党政一把手在招商中的作用。力争全年实际利用外资5.7亿美元，引进内资突破135亿元。五是提高外经外贸水平。选择10家龙头出口企业和10家具有发展潜力的中小型出口企业重点扶持，力争培育出口超亿美元企业3家，3－5个出口产品列入国家和省出口品牌，山船修造船基地成为国家级出口基地。积极实施“走出去”战略，鼓励企业开展境外投资、承揽国际工程、建设海外园区。

（八）加强社会管理与公共服务，切实保障和改善民生。一是千方百计扩大就业。强化政府投资、重大项目建设与增加就业联动机制，重点解决好高校毕业生、农村转移劳动力和城镇就业困难人员就业创业问题，确保城镇新增就业4.7万人、农村劳动力转移就业3.5万人、应届高校毕业生当年就业率达到80%以上。推进创业型城市建设活动，力争创业带动就业1万人以上。深入推进小企业劳动合同3年行动计划，积极营造和谐劳动关系。二是健全社会保障体系。以非公有制单位从业人员、灵活就业人员、在校大学生、城镇困难群体和农民工参保为重点，扎实做好各类社会保险扩面和管理工作。扩大城镇居民养老保险和新农保试点工作。实施社保卡“一卡通”便民工程，稳步推进医疗保险市级统筹工作。进一步提高城乡低保标准，城市区达到每月290－330元，农村达到每年1600元。实施扶贫开发“221工程”，确保2000户养殖户增收，培训致富能手2000人以上，1万人稳定脱贫。加强保障性安居工程建设，新建廉租住房1600套、经济适用住房2300套、公共租赁住房5200套、限价商品住房2000套。三是全面发展各项社会事业。推进义务教育均衡发展，加快中小学标准化建设、薄弱学校改造，新改扩建中小学校舍26万平方米。加强教师队伍培训。大力发展以农村为重点的职业教育。扩大学前教育资源，提高高中教育水平，支持高等教育发展。扎实推进医药卫生体制五项重点改革。新农合筹资标准提高到210元。公办乡镇卫生院、社区卫生服务中心全部实施基层药物卫生综合配套改革。完成市中医院、昌黎县医院、抚宁县医院、卢龙县医院迁建改建工程。推进市精神卫生中心、市第三医院重点传染病医疗救治中心建设。加强乡镇卫生院、村卫生室、社区卫生服务中心标准化建设。做好传染病防治工作。启动市博物馆、科技馆、规划馆项目。加快市青少年宫建设。建成乡镇综合文化站9个、文化信息资源共享工程村级服务点236个、农家书屋726个。深入实施全民健身工程，完成100个行政村和社区健身路径建设，积极承办大型体育赛事。四是维护社会安全稳定。开展食品安全监管长效机制建设落实年和药品安全规范年活动。加强葡萄酒、水产品、玉米罐头、淀粉及制品、熟食制品等重点领域专项整治，依法严厉打击制假售假行为，规范行业秩序，不断提高安全食品市场占有率。严格落实安全生产责任制和承诺制，推进企业安全质量标准化，持续开展安全生产执法检查活动，努力杜绝重特大事故的发生。加强社会治安综合治理，排查化解矛盾纠纷，建设平安秦皇岛。加强突发公共事件预防及应急反应机制建设，提高应急救援能力。强化“大暑期大北戴河”理念，举全市之力打好“16+3”暑期重点工程大会战，确保暑期前城市形象面貌有新的改善和提升，倾力打造精品北戴河、魅力北戴河，提高暑期服务保障水平。

加强基层民主政治建设。统筹做好人口计生工作。进一步做好国防动员、人民防空、民兵预备役工作，开展双拥共建活动。支持工会、共青团、妇联、科协等群众组织开展工作。发展老龄、残疾人和慈善事业。推动司法行政、民族宗教、外事侨务、气象、防震减灾、地方志、档案等各项事业全面进步。

（九）强化政府自身建设，努力打造人民满意的政府。“十二五”宏伟目标对政府工作提出了新的更高要求。我们必须以奋发有为的精神和姿态、昂扬向上的激情和斗志、争创一流的信心和勇气，认真履职尽责，勤勉敬业。一是进一步增强政府创新能力。坚持对上负责与对下负责高度统一，贯彻上级决策部署与工作实际有机结合，创新发展理念、发展模式、发展举措，积极破解改革建设中遇到的热点难点问题；抢抓系列机遇，努力把各种资源更好配置到对经济社会发展具有决定作用的领域和环节，使经济增长建立在优化结构、提高效益、降低消耗、保护环境、改善民生的基础之上。二是进一步提高依法行政水平。坚持重大问题集体讨论决定，完善公众参与、专家咨询的决策机制，不断提高决策科学化、民主化水平。全面落实人大及其常委会的决议决定，主动接受监督并报告工作。自觉接受政协民主监督，广泛听取社会各界的意见和建议，主动接受新闻舆论和人民群众的监督。健全权责一致的执行机制，推行行政问责制和绩效评估制，提高政府执行力和公信力。三是进一步改进工作作风。加强公务员队伍建设，强化公仆意识，千方百计为群众、为基层、为企业办实事、解难事。规范市县乡三级政务服务标准，实质性提高服务质量和效率。继续精简会议、文件，改进会风、文风。发挥好政风行风热线作用，做好民主评议工作。四是进一步加强廉政建设。严格落实廉洁从政各项规定。完善“机制+科技”防治腐败模式，加强专项资金监控、项目统一招投标、廉政勤政预警、政府采购等平台建设，建立健全预防和惩治腐败的长效机制，着力从源头上防治腐败。严肃查处违法违纪案件，切实纠正损害群众利益的不正之风。

各位代表！回顾过去，我们豪情满怀；展望未来，我们信心百倍。让我们高举中国特色社会主义伟大旗帜，紧紧团结在以胡锦涛同志为总书记的党中央周围，在省委省政府和市委的正确领导下，上下一心、团结奋进，努力创造出经得起历史、实践、群众检验的实绩，把我们的家园建设得更加美好！
